# Riga Fede Disease

**DOI:** 10.21699/jns.v6i1.475

**Published:** 2017-01-01

**Authors:** Pratej Kiran Kanumuri

**Affiliations:** Panineeya Institute of Dental Sciences, Hyderabad

**Dear Sir**

A 4-day-old male baby presented with congenitally erupted two teeth on lower jaw since birth resulting in difficulty in breast feeding. Oral examination showed ulceration (size 1cm x1cm) on the ventral side of the tongue with two natal teeth on lower mandibular anterior region (Fig.1A). Radiographic examination revealed normally developing primary teeth germs below the erupted teeth like structures (Natal teeth) (Fig. 1B). Prophylactic administration of vitamin K (0.5mg, IM) was done. Topical anaesthetic gel was applied and teeth were extracted. Extracted teeth showed well developed crown and root. (Fig. 1C) After one week completely healed exraction socket and site of ulcer was observed. Parent felt comfortable while feeding and even child was comfortable in suckling.

**Figure:**
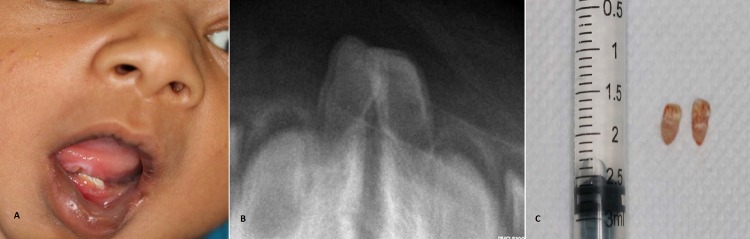
Figure 1: A) Natal teeth seen in the lower arch and ulcer seen on the ventral side of tongue. B) Intraoral radiograph showing two natal teeth in mandibular anterior region. C) Extracted Natal teeth.

Natal teeth are those observable in the oral cavity at birth and neonatal teeth are those that erupt during the first 30 days of life.[1] Incidence ranges from 1:2000 to 1:3500 births.[2] Exact etiology is unknown, it is thought be due to superficial portion of the tooth germ, trauma, malnutrition, infection, hormonal stimulation, febrile states, and maternal exposure to environmental toxins. These teeth are frequently found associated with syndromes. Complications related to natal and neonatal teeth include irritation, discomfort during suckling, and trauma to infants' tongue, sublingual ulceration (Riga-Fede disease), laceration of the mother's breast and chance of aspiration of the mobile natal teeth [3] this might be the reason for extraction of mobile teeth. Extraction of natal teeth should ideally be delayed for 10 days after birth to avoid haemorrhage due to hypoprothrombinaemia; before 10 days it can be extracted after administering Vit. K.

## Footnotes

**Source of Support:** Nil

**Conflict of Interest:** None
